# Supplementary material for: A Comprehensive Assessment of Ultraviolet-Radiation-Induced Mutations in Flammulina filiformis Using Whole-Genome Resequencing
Source: J Fungi (Basel). 2024 Mar 20;10(3):228. doi: 10.3390/jof10030228 (PMC10971301; doi:10.3390/jof10030228)
Supplement: Supplementary file 1 [file jof-10-00228-s001.zip › Supplementary Material S8/KEGG annotation/out/64381550635650.os/KO/out_map/map05323.html]

KEGG PATHWAY: Rheumatoid arthritis - Reference pathway


|  |  |
| --- | --- |
| **Rheumatoid arthritis - Reference pathway** |  |

[
Pathway menu
| Organism menu
| Pathway entry
| Show description
| User data mapping
]

Rheumatoid arthritis (RA) is a chronic autoimmune joint disease where persistent inflammation affects bone remodeling leading to progressive bone destruction. In RA, abnormal activation of the immune system elevates pro-inflammatory cytokines and chemokines levels, which can promote synovial angiogenesis and leukocyte infiltration. The synovium forms a hyperplastic pannus with infiltrated macrophage-like and fibroblast-like synoviocytes and invades joints by secreting proteinases and inducing osteoclast differentiation.

|  |  |
| --- | --- |
| Reference pathway | 100% |
